# Implementing Culturally Informed Dementia Risk Reduction Strategies in First Nations Communities: A Continuous Quality Improvement Approach

**DOI:** 10.1002/alz70860_098949

**Published:** 2025-12-23

**Authors:** Rachel Quigley, Sarah G Russell, Yvonne Hornby‐Turner, Edward Strivens

**Affiliations:** ^1^ James Cook University, Cairns, QLD, Australia

## Abstract

**Background:**

Dementia risk is up to five times higher in First Nations communities, necessitating a culturally informed response through primary health interventions. Addressing this disparity requires strategies that are both culturally safe and tailored to community needs.

**Method:**

A decolonizing approach was employed to identify and implement culturally appropriate dementia risk reduction strategies in one urban and three rural communities in Queensland, Australia. Aboriginal Participatory Action Research (APAR) and Continuous Quality Improvement (CQI) methodologies were used to guide this study. Data were gathered through research yarning with stakeholders, including community members, primary health care (PHC) staff, and service providers. Health records were audited, and existing clinical processes and services were mapped to inform intervention strategies. Interventions were customized to each community based on feedback and priorities, with CQI cycles conducted over three years using the Plan‐Do‐Study‐Act model. Project evaluation focused on process, impact, and sustainability using the RE‐AIM framework.

**Result:**

Four dementia‐related goals were actioned within the CQI framework: i) increasing clinic‐based screening and assessment rates for cognitive impairment and dementia; ii) enhancing staff capacity to deliver best‐practice primary health care for the prevention and management of dementia‐related risk factors; iii) empowering communities to recognize and respond to dementia risk through training and education; and iv) co‐developing preventive health and health promotion strategies to address dementia risk in the broader Indigenous population. Indigenous leadership, key‐skills working groups, and co‐development with health practitioners and community members were central to achieving these goals.

**Conclusion:**

The iterative engagement of health practitioners and community members facilitated the translation of current knowledge into practice, with CQI outcomes becoming embedded in health service delivery. This culturally tailored approach has demonstrated its potential to improve dementia‐related care and prevention in First Nations communities, offering a model for other regions facing similar challenges.

